# An Opportunity to See the Heart Defect Physically: Medical Student Experiences of Technology-Enhanced Learning with 3D Printed Models of Congenital Heart Disease

**DOI:** 10.1007/s40670-023-01840-w

**Published:** 2023-08-01

**Authors:** Jack C. Luxford, Tegan L. Cheng, Jonathan Mervis, Jennifer Anderson, Jillian Clarke, Sarah Croker, Erez Nusem, Liam Bray, Hasantha Gunasekera, Karen M. Scott

**Affiliations:** 1 Faculty of Medicine and Health, Children’s Hospital Westmead Clinical School, The University of Sydney, Sydney, NSW Australia; 2 Heart Centre for Children, The Children’s Hospital at Westmead, Sydney, Australia; 3 Sydney School of Health Sciences, The University of Sydney, Sydney, NSW Australia; 4 EPIC Lab, The Children’s Hospital at Westmead, Sydney, Australia; 5 Discipline of Medical Imaging, Faculty of Medicine and Health, The University of Sydney, Sydney, NSW Australia; 6 School of Medical Sciences, Faculty of Medicine and Health, The University of Sydney, Sydney, NSW Australia; 7 School of Architecture, The University of Queensland, Brisbane, QLD Australia; 8 Faculty of Architecture, Design and Planning, The University of Sydney, Sydney, NSW Australia

**Keywords:** 3D printing, Technology-enhanced learning, Medical education, Congenital heart disease

## Abstract

Three-dimensional (3D) printing is increasingly used in medical education and paediatric cardiology. A technology-enhanced learning (TEL) module was designed to accompany 3D printed models of congenital heart disease (CHD) to aid in the teaching of medical students. There are few studies evaluating the attitudes and perceptions of medical students regarding their experience of learning about CHD using 3D printing. This study aimed to explore senior medical students’ experiences in learning about paediatric cardiology through a workshop involving 3D printed models of CHD supported by TEL in the form of online case-based learning. A mixed-methods evaluation was undertaken involving a post-workshop questionnaire (*n* = 94 students), and focus groups (*n* = 16 students). Focus group and free-text questionnaire responses underwent thematic analysis. Questionnaire responses demonstrated widespread user satisfaction; 91 (97%) students agreed that the workshop was a valuable experience. The highest-level satisfaction was for the physical 3D printed models, the clinical case-based learning, and opportunity for peer collaboration. Thematic analysis identified five key themes: a variable experience of prior learning, interplay between physical and online models, flexible and novel workshop structure, workshop supported the learning outcomes, and future opportunities for learning using 3D printing. A key novel finding was that students indicated the module increased their confidence to teach others about CHD and recommended expansion to other parts of the curriculum. 3D printed models of CHD are a valuable learning resource and contribute to the richness and enjoyment of medical student learning, with widespread satisfaction.

**Supplementary Information:**

The online version contains supplementary material available at 10.1007/s40670-023-01840-w.

## Introduction

The technology of three-dimensional (3D) printing has undergone a dramatic evolution in recent years, with rapid uptake across the healthcare industry [1]. 3D printing has been demonstrated to be a valuable educational tool in the teaching of general and cardiac anatomy for both science [2] and medical students [3–5]. These studies have demonstrated superiority or non-inferiority to traditional cadaveric or two-dimensional (2D) teaching modalities in the areas of knowledge acquisition and learner satisfaction [6, 7]. A recent systematic review and meta-analysis of the use of 3D printed resources compared to conventional teaching modalities in medical education found that 3D printed resources were superior in terms of learner satisfaction and short-term knowledge acquisition [8]. However, for the sub-group of five studies that specifically looked at cardiac anatomy, there was no significant difference in post-teaching knowledge scores between conventional and 3D printed anatomy teaching. A contemporary survey of Polish medical and health-science students [9] found that a majority (79%) were willing to participate in medical education activities related to 3D printing.

Congenital heart disease (CHD) is the most common congenital anomaly, occurring in 1% of live births [10] and will be encountered by family medicine, emergency, paediatric, and adult physicians across the spectrum of their clinical practice. Adult CHD is estimated to affect 3000 in every 1 million adults and this proportion is likely to increase with increased CHD survival rates [11]. Inadequate clinician knowledge can lead to suboptimal care of these patients for both simple and complex lesions [12], and thus this is an important area of medical education. Recent efforts have been made to introduce 3D printed models of CHD teaching across a spectrum of learners including undergraduate nursing [13] and medical students [6, 7, 14–16]; paediatric [17, 18], critical-care [19–21], and cardiac surgical and physician trainees [22–24]; and adult and paediatric cardiac nurses [25]. These studies have demonstrated the feasibility and some efficacy in the use of 3D printed models of CHD as learning aids, but have not comprehensively explored students’ attitudes and experiences with these novel learning aids. Additionally, there has been little focus on *how* learners are directed or facilitated to use 3D printed models, and thus how they can be incorporated into new or existing curricula. We have previously demonstrated that students given an unstructured opportunity to explore both waxed cadaveric specimens and 3D printed models of CHD overall spend an equivalent time holding and interacting with both models — and tend to interact more with pipe-cleaners and more robustly handle the 3D models [26]. Medical education has rapidly adopted technology-enhanced learning (TEL) over the past decade [27], especially in the field of anatomy [28]. However, TEL as it pertains to congenital cardiac anatomy has not previously been evaluated.

In late 2019, as part of an interdisciplinary group, the authors designed a cardiac pathology workshop incorporating a self-directed online module with guided exploration of online and 3D printed models of congenital cardiac disease. This self-directed workshop replaced a previous 30 minute in-person session with didactic teaching on paediatric cardiac pathology using waxed cadaveric specimens of CHD. The cadaveric specimens were part of a regular teaching set housed at The Children’s Hospital at Westmead, NSW, Australia, which were at risk of being degraded over time with repeated use. Students at the University of Sydney Medical Program complete their paediatric teaching across metropolitan and regional hospitals, and students at regional hospitals were unable to access these centrally housed specimens. The emergence of the COVID-19 pandemic further provided stimulus for the redevelopment of this practical session due to concerns around adequate cleaning of specimens between all students, and the need to limit large group interactions.

We created a small-group based, self-directed cardiac pathology workshop to enhance student confidence and competence in understanding and applying knowledge related to CHD. This cardiac pathology workshop used online clinical cases and digital 3D models to facilitate a guided exploration of identical hand-held 3D printed models of anatomic specimens. Inspiring confidence and self-efficacy was key, insofar as the complexity of CHD and its perception as difficult or confusing to medical students can lead to disillusionment and abandonment of the subject area. This reflects Bandura’s concept of self-efficacy in social cognitive learning theory, as a student’s conception of their self-efficacy influences goal selection, effort investment, and resilience in the face of adversity and difficulty [29].

This study aimed to explore medical students’ perceptions of the use of 3D printed models of CHD, and assess their self-efficacy and confidence in knowledge about CHD following a self-directed CHD workshop incorporating online and 3D printed models of CHD.

## Materials and Methods

### Setting

The University of Sydney Medical Program is a graduate entry 4-year medical degree. At the time of the study, students in third or fourth year completed a compulsory 8-week Child and Adolescent Health Specialty Block, which includes curriculum topics focussed on CHD. Cardiac teaching in the Medical Program includes didactic lectures in the first two years and a lecture and interactive cardiac pathology workshop during the Child and Adolescent Health Block. This workshop was delivered four times annually either at the Children’s Hospital at Westmead (a tertiary/quaternary paediatric hospital) or at one of the rural sites (Orange, Dubbo, and Lismore Base Hospitals).

### Workshop

The cardiac pathology workshop was designed around four clinical cases of children with CHD. It involved a 45-min in-person self-directed mixed-media session incorporating a digital module and hand-held 3D printed models of CHD, supported by a single facilitator. Facilitators did not have specialist paediatric cardiology expertise, and included junior paediatric trainees and adult cardiologists, in a ratio of 1 facilitator to 25 students. A 5-min online lecture on the fetal circulation was available for students to watch prior to the workshop. The learning objectives of the workshop were for students to be able to identify normal and abnormal features of cardiac anatomy and CHD, and to relate these features to cardiac physiology and their clinical implications.

The digital module was built in collaboration with three computing design under-graduate students at the University of Sydney, supervised by LB. Key features in the design included simple navigation, a capacity to move through individual clinical cases sequentially, and a 3D rendering (i.e. digital 3D image) of the identical hand-held model the students would use in-person. The online module was designed to be 'clicked through' in a way that would guide students to match their hand-held model with the online 3D model, thus providing a semi-structured guided exploration of the 3D models. This online 3D model could be zoomed into and rotated, and have labels removed and added to enhance clarity.

**Fig. 1:**
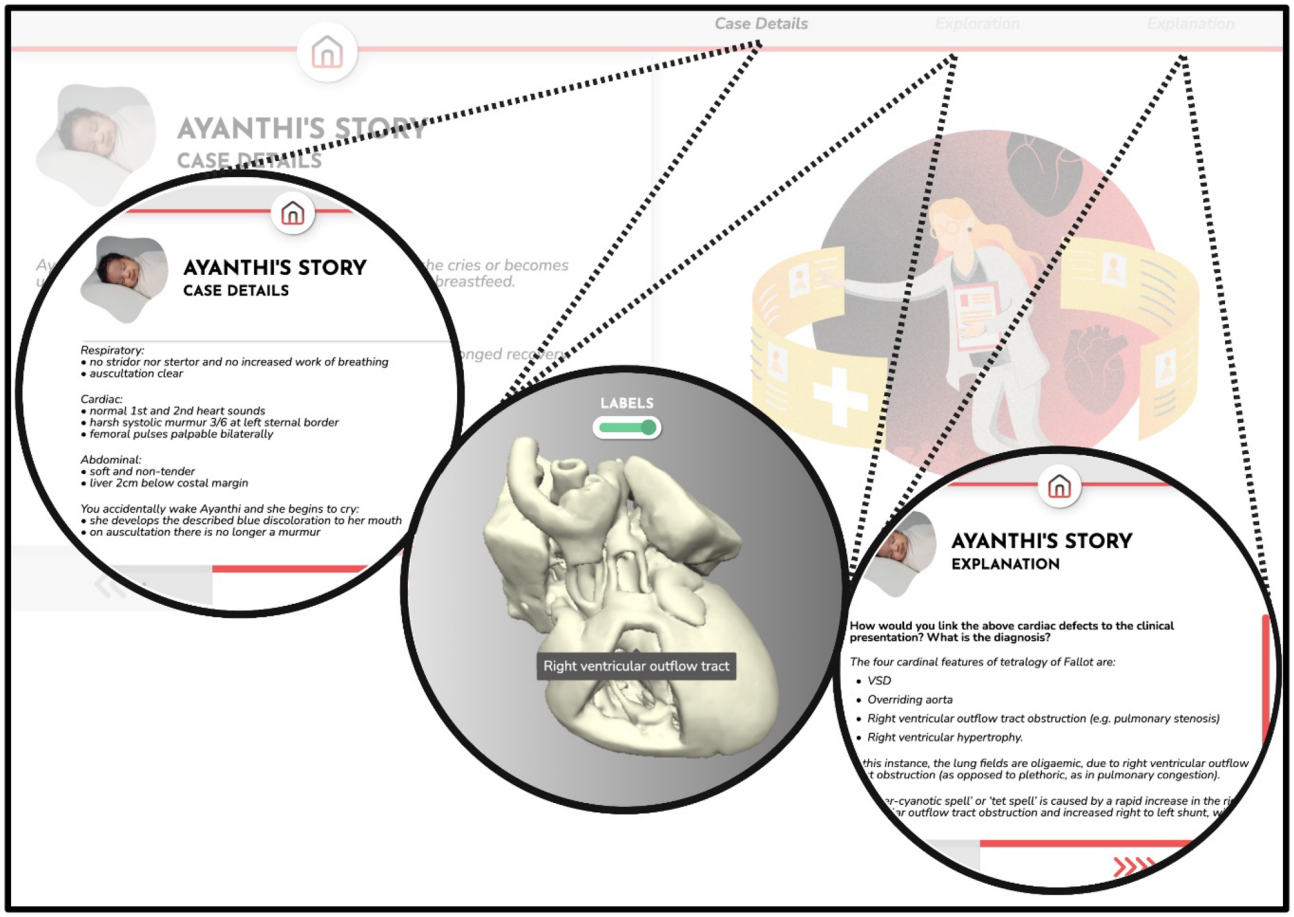
An example of a case of tetralogy of Fallot from the technology-enhanced learning module. Each clinical case initially presented the history, examination findings, and a chest x-ray. The next stage of the case was the virtual 3D heart model, which guided students through their exploration and discovery of the physical 3D heart. The virtual model could be rotated and enlarged, and included the option of labels. The final stage was an explanation of the pathophysiology of the case to consolidate learning

**Fig. 2:**
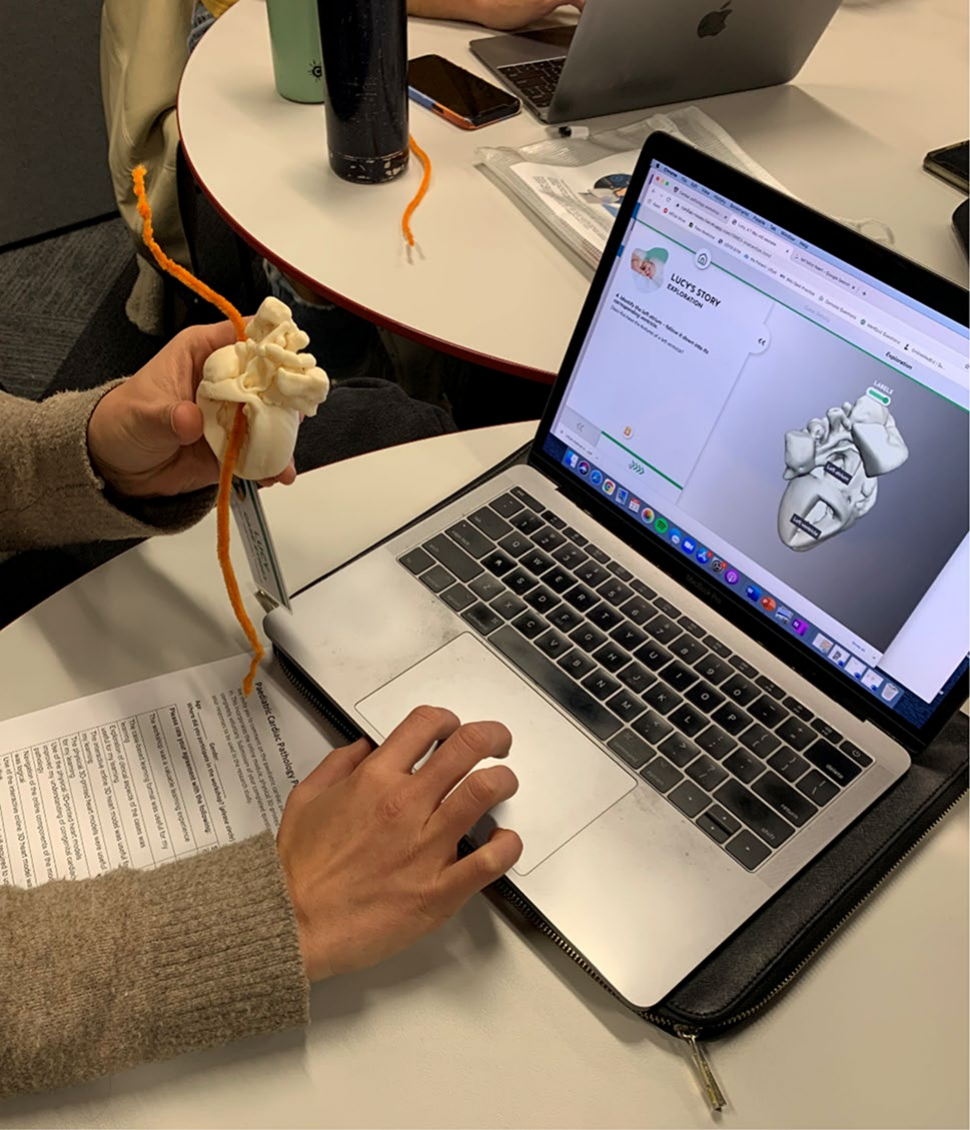
A student working their way through the workshop with the 3D model, pipe-cleaner, and technology-enhanced learning module

In small self-directed groups of two or three, students worked through the four unfolding clinical cases of CHD in the online module: ventricular septal defect with patent foramen ovale, patent ductus arteriosus, coarctation of the aorta with an atrial septal defect, and tetralogy of Fallot. Each case had three components (see Fig. 1). The first was the clinical case, with details of the presenting symptoms, examination details, and chest radiographs; students were prompted to discuss with their group the relevant cardiac and non-cardiac differential diagnoses as they worked through each stage of the case. The second component of the online module then led the students through a digitally facilitated exploration of the 3D printed model of the specific heart lesion, using pipe-cleaners, paper-clips, and mobile telephone lights to explore and understand the anatomic and physiologic substrate that contributed to the case presentation (see Fig. 2). The online 3D model was identical to the 3D printed model that students held, which aided in students’ orientation and identification of normal and abnormal cardiac anatomy. Finally, students were provided with an explanation of the underlying cardiac physiology of the case. The online module remained available to students after the workshop to revise and consolidate their learning.

### Three-dimensional Printing

**Fig. 3:**
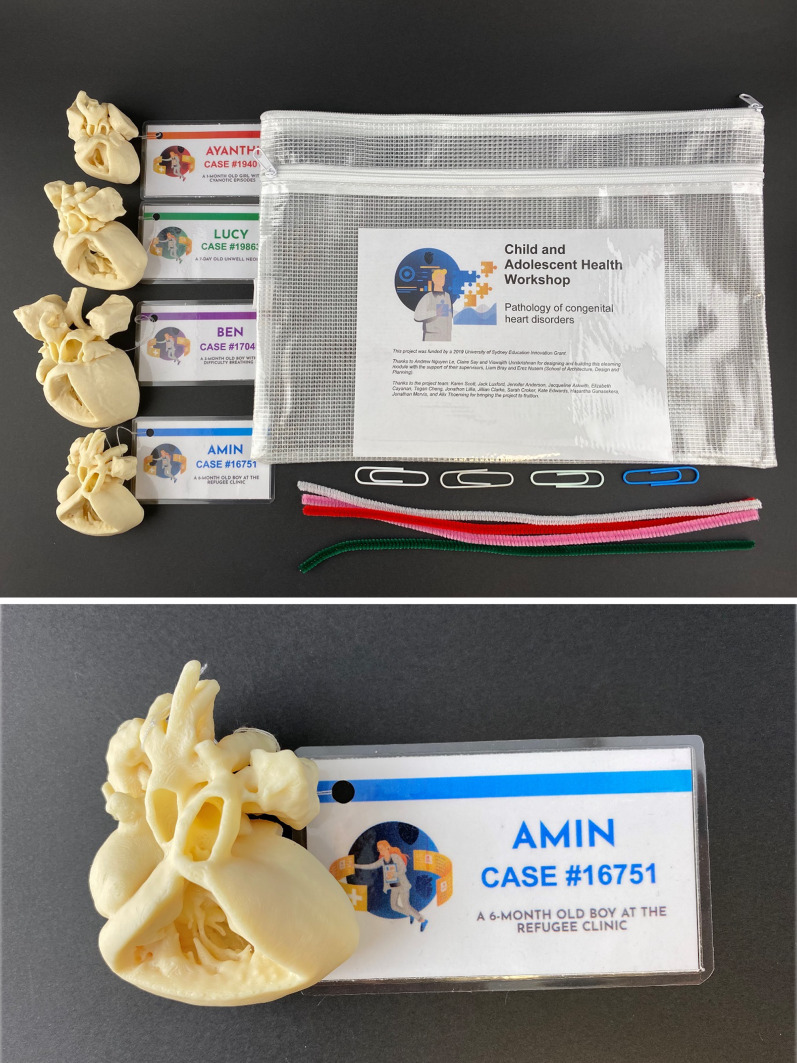
**a** The cardiac pathology workshop equipment including four 3D printed models of congenital heart disease. **b** A case of patent ductus arteriosus

3D printed heart models were produced from computer tomography (CT) scans (0.4 mm slices, Siemens SOMATOM Force, Munich, Germany) of existing, anonymised, fixed, and waxed cadaveric heart specimens from the Department of Pathology, The Children’s Hospital at Westmead. Four specimens were chosen as representative of simple and complex CHD — ventricular septal defect with patent foramen ovale, patent ductus arteriosus, coarctation of the aorta with an atrial septal defect, and tetralogy of Fallot. CT scans were exported as DICOM files, segmented using Mimics Innovation Suite (v19.0, Materialise, Leuven, Belgium), and 3D printed at scale (see Fig. 3) in acrylonitrile styrene acrylate (ASA) using material extrusion (Fortus 450mc, Stratasys, Israel).

### Study Design

This was a concurrent triangulation mixed-methods study [30] involving a questionnaire and focus groups. The questionnaire examined the students’ attitudes and perceptions towards 3D printed models of CHD, the online module, and their self-efficacy in learning about paediatric cardiac pathology. The focus groups further explored students’ experience in using the 3D printed models to identify novel insights into student engagement with 3D printed models of CHD and the digital module.

### Recruitment and Participants

Participants were recruited from third and fourth year medical students who attended the cardiac pathology workshop in 2021. Attendance at this non-graded workshop was compulsory, but participation in the study (questionnaires or focus groups) was voluntary. Administrative staff informed students about the study via email before the workshops. The workshop facilitator and focus group interviewers were not involved in student assessment.

### Data Collection

At the end of the workshop, students were provided with a hard-copy of the anonymous questionnaire (see Appendix A), which took approximately 10 min to complete. The questionnaire was a modification of the Cook and Ellaway [31] short learner evaluation of TEL. It entailed 14 questions using a 7-point Likert scale (strongly agree to strongly disagree) and five free-text questions regarding engaging aspects of the workshop and suggestions for improvement. Participants were also asked to indicate their gender, age, and location of the workshop (urban or rural).

Two 30-min focus groups were held with small groups of eight students each, within 1 week of the workshop. The audio-recorded focus groups were conducted by two interviewers (JL and KS) via video-conferencing using a series of discussion questions (see Appendix B). JL led the questions and discussion, whilst KS took field notes. Focus groups were repeated until theoretical saturation was achieved (at two).

### Data Analysis

Individual questionnaire responses were collated and stored in an Excel spreadsheet. Demographic details were summarised using frequency counts and percentages for categorical variables and mean and standard deviation (SD) for continuous normally distributed variables. Missing responses were excluded listwise from analysis. Analysis was performed with GraphPad Prism v9 (GraphPad Software, San Diego, CA) and figures were created with BioRender.com.

Audio-recordings of the focus groups were transcribed and the recordings then deleted after consultation with field notes. Focus group transcriptions and qualitative questionnaire responses were analysed in duplicate (JL and TC) using thematic analysis [32]. Data were coded line by line to form themes and sub-themes. A coding table was created to classify all data, and differences were discussed amongst the researchers (JL, TC, EN, and KS) until consensus on themes was reached.

## Results

### Questionnaire Responses

**Fig. 4:**
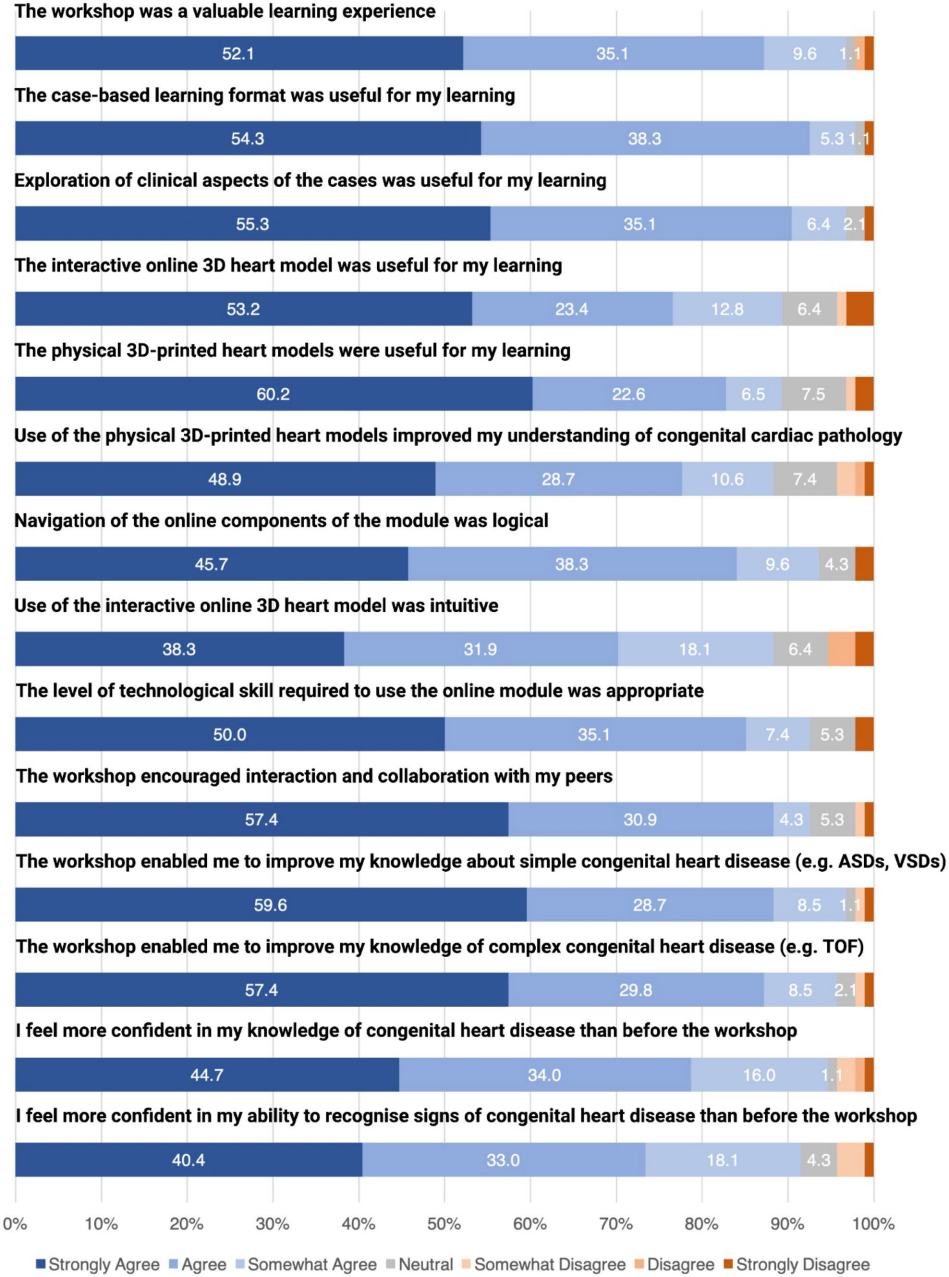
Questionnaire responses

Two-hundred and twenty students attended the cardiac pathology workshop between January 2021 and July 2021. Of these, 94 (43%) submitted a completed questionnaire. There were 47 (50%) females, 43 (46%) males with 4 (4%) respondents not stating a gender. Mean age was 27.7 (SD ± 2.9) years, with five respondents not stating their age. Seventy-five (80%) respondents attended the workshop at The Children’s Hospital at Westmead and 19 (20%) at one of the three regional hospitals. All questions were completely answered except for one missing response to question five. Quantitative responses to the questionnaire are reported in Fig. 4.

### Thematic Analysis

**Fig. 5:**
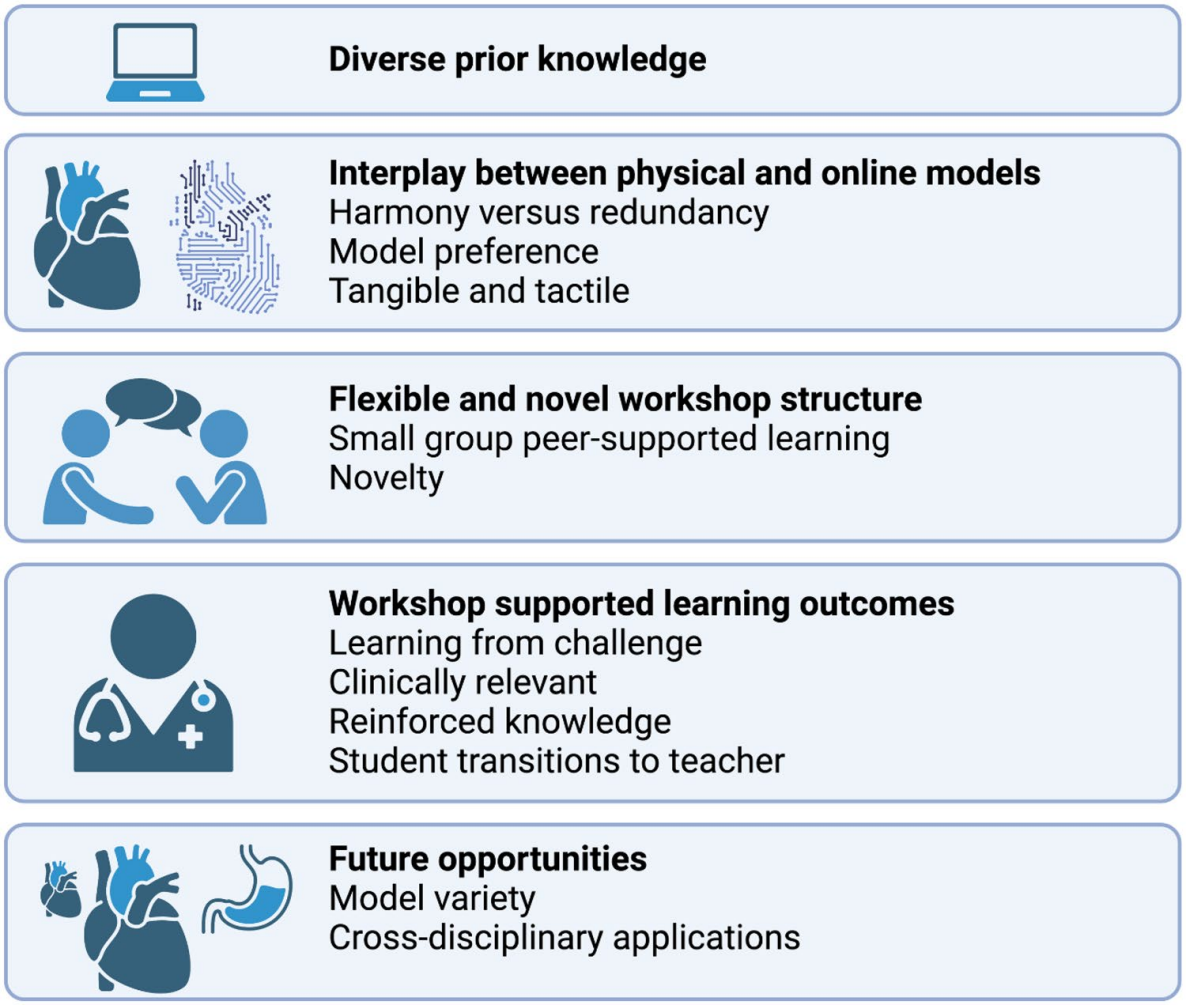
Diagrammatic representation of thematic relationships

Sixteen students participated in two focus groups. Free-text responses to five questions in the questionnaire (Appendix A) were combined with the focus group discussion for thematic analysis. Five major themes were identified from the qualitative data, with twelve sub-themes. Thematic relationships are displayed graphically in Fig. 5. Students brought (A) variable prior knowledge regarding CHD to the workshop, and utilised the (B) interplay between the physical and online models in (C) a flexible and novel workshop structure. The workshop (D) supported their learning outcomes, and students identified (E) opportunities for growth.

### Diverse Prior Knowledge

Students had variable knowledge of CHD prior to the workshop, despite having participated in the same early medical school lectures. Some students reported a good pre-existing knowledge, and that “*based on the lectures we’d done here and there, we were adequately prepared but I feel like even if in theory you weren’t, you would still be fine*”. These prior experiences included more formal didactic pre-clinical lectures, and cadaveric anatomy teaching using normal hearts. However, multiple students felt that previous teaching had left them bewildered, commenting that historically they had received “*a lot of very didactic lectures where there’s all this info and trying to remember it*”. One student reflected that, unlike their former teaching, the cardiac pathology workshop provided “*an opportunity to see the heart defect physically, and not just close your eyes and try to visualise it like in first year*”.

### Interplay Between Physical and Online Models

#### Harmony vs. Redundancy

One of the key aims of the online module and use of the virtual 3D heart was to orient and guide the students through the exploration of the 3D printed model, to mitigate the confusion that can otherwise be felt in a self-directed anatomy session. Most students found that the hand-held and online models complemented each other in a harmonious fashion, where the online model facilitated a structured exploration of the physical model. Students said, “*We sort of used them together so I think the physical model was great, probably preferred that but then the online one was really helpful to orientate yourself to the physical one*”. The labels on the online model “*were really good, they were probably the only thing that really helped with orientation*”. However, some students struggled to identify the anatomy without the tutor’s assistance, even with the online orientation, commenting that “*we spent quite a bit of time on the outflow tracts and I don’t think the labels really helped me. It just took us a long time to find them*”. A few students found that there was unnecessary repetition: “*there was quite an overlap and the hand-held models would have been enough*” or that the models were interesting, but not essential, to their learning: “*They were definitely cool, I just think if they were like missing from the session, it wouldn’t really have impacted my learning*”.

#### Model Preference

Some students expressed a clear preference for one modality (hand-held or online) compared to the other, often with significant enthusiasm. One student said, “*I liked the hand-held models over the ones on the website just because it was way easier to just hold something and move it exactly how you want it*”. This contrasted with another’s suggestion for improvement: “*Not making me come play with plastic toys. This could have been done ONLINE*”. This reflected unique learning preferences, and students were often clear and articulate about their learning needs, saying “*I much preferred the physical models over the virtual – very interesting*”. Whilst some students had a clear preference, the availability of both models in the workshop meant that these could be complementary modalities without compromising an individual’s time nor learning preferences, and some students reflected on this: “*I prefer the hand-held ones but …if I didn’t have those handy, then I think having it on the website kind of supplements and would supplement my learning*”.

#### Tangible and Tactile Interactions

Medical students voiced significant enthusiasm for the tactile and concrete interactions with the 3D printed models of CHD. Students found that being able to touch, feel, and see the heart defects made it easier to understand both the defects and the relationships between structures, and said “*There [were] a couple of things that were easy to appreciate—the relationship between chambers and how big they are and that kind of stuff. It developed us and made us appreciate that better*”. The relationship between anatomy, physiology, and pathophysiology was made clearer, and one student commented “*I think just having the pipe cleaners and stuff to stick in the different defects was fun, a different way to sort of appreciate it and not just the big gaping holes*”. Graduate medical students are mature learners, aware of their learning preferences and able to articulate this, for example: “*As a visual learner, I really enjoyed the models*”. Some felt they were already confident in the material and subject matter, but that “*You get a more detailed perspective on how small the hearts are and how small the defects can be, as well as seeing I guess ventricular hypertrophy or stuff like that in front of you*”.

### Flexible and Novel Workshop Structure

#### Small Group Peer-Supported Learning

The structure of the module was built to be largely self-directed, and encourage peer-collaboration without significant specialised instructor knowledge or involvement. Students were broadly positive and found the online learning module well-made and easy to navigate, complementing the case-based learning in small groups. One said “*we felt really lucky that we had a small group so we could sort of spend as much time and ask all sorts of questions about things which was good*”. The non-didactic workshop format was preferred to traditional lectures: “*We found it useful to have it with cases online so that it wasn’t just the normal lecture format we were used to and we could have a more practical component to it*”. Despite the intention that the workshop was largely self-driven, students still reported significant interactions with the tutors available: “*we did have a tutor run through it with us which was good and probably helped fill in the blanks in terms of what was going on behind the scenes*”. Students particularly enjoyed the opportunity for collaboration, with their peers and with the facilitators, as they engaged in the problem-solving of unpacking the clinical case.

#### Novelty

Students repeatedly described the workshop as fun, and this was reflected in the vigour and enthusiasm of some responses. The theme of play was also repeated in feedback, as students conceived of the cases as games and puzzles to be solved, rather than strictly academic pursuits. Students found the workshop creative and innovative and novel, commenting “*It’s novel compared to the other workshops and teaching that we have and it’s like different from going through a textbook or an online resource*”. Students reflected that they appreciated things they hadn’t anticipated or understood previously: “*We all know we’ve got two atria and two ventricles, like that’s not breaking news, but just sort of seeing how the effects manifest and whatnot was really cool*”.

### Workshop Supported Learning Outcomes

#### Learning from Challenge

The workshop challenged the students in a constructive manner to learn from real examples of CHD. Students enjoyed the clinical challenge of deduction, and the puzzle-solving nature of case-based learning and the difficulties entailed in diagnosing CHD. Students appreciated the challenge, for example: “*I liked the fact the models weren’t labelled… otherwise I would have just relied on the labels that were there and I wouldn’t really try and figure it out and orientate it*”. Despite the challenges, they felt the workshop “*helped to guide us on what we didn’t feel so comfortable with*”, ensuring there was a safe mechanism to progress learning even when they encountered obstacles.

#### Clinically Relevant

Students particularly felt that the case-based and clinically oriented aspects of the workshop were rewarding and useful for their learning. Students commented “*I liked the human component, like as an actual case, that was very helpful*”. They felt this actively challenged them to use their clinical reasoning, think laterally and flexibly, and could imagine real-life situations where they could put this knowledge into practice. One student said, “*I think anything that has a clinical focus that is a bit more involved where you’re [using] your hands and you can think in a group is always going to be preferable to didactic learning*”. They enjoyed the dynamic of the two models (online and hand-held) as it gave them an “*opportunity to diagnose and then re-affirm the diagnosis with the heart model*” and predicted that “*these sorts of things can present whenever, you know it’s not just baby checks but presenting weeks or a few years later, whether you are working in emergency or general practice or paediatrics*”.

#### Reinforced Knowledge

The practical workshop enabled the students to link in their previous knowledge and understanding of CHD and prepared them for examinations and assessments. One student said “*The general style of teaching using models and being able to visualise it helped a lot… anything that is anatomical if you can have a model to visualise it I personally take a lot more out of [it]*”. Even students who felt confident could take value from the session, for example: “*Even though I felt I had a good understanding, I felt it just solidified and also any activity that we do which is memorable, makes it more likely you are going to recall something and understand it*”. The digital module and the physical 3D printed models enhanced the clarity of their understanding, and students liked the opportunity to “*access it again at home (no pressure)*”. Students felt because of the workshop they had an improved understanding of cardiac anatomy and pathophysiology in relation to CHD.

#### Student Transitions to Teacher

The adage of 'see one, do one, teach one' is long-standing in clinical medicine, and students volunteered that they felt the workshop had increased their understanding of CHD, and thereby their confidence to act as future educators. One student reflected that “*pretty much anywhere you work you are probably expected to have some teaching role for students and junior doctors, and I think having been able to visualise these congenital defects, it makes it much easier to be able to teach it in the future*”. Another commented that “*If we are ever teaching medical students in a cardiology term about these things, I think we’ve got these models to be able to have a good concept of what they are*”. The workshop increased students’ confidence both as learners and future teachers.

### Future Opportunities

#### Model Variety

Whilst students overwhelmingly felt the 3D models were useful and important to their understanding of CHD, some had suggestions for changes to future 3D model learning. There was disagreement between students on the optimal scale and size of the models. Some students appreciated the to-scale replication of the defects “*it was cool to be able to see it life-sized and to really appreciate that these septal defects or whatever might not actually be that big, and can be quite subtle*” whilst others felt like this was a hurdle they could not surmount “*It was hard to see everything sometimes, because obviously, they are very small because the kids are very small, but we were kind of like, ‘Oh – is there a hole there?*” Students agreed it might be best to have both, “*a big model that’s very obvious and not very challenging and then a life-sized model so you can do that conversion of appreciating the detail*”. Some students felt a normal 3D printed heart would be useful to help with orientation and learning to become comfortable with the models. Additional proposals included the idea of colour-coding, to identify normal and abnormal features, and to develop features that reflect blood flows in the abnormal heart models.

#### Cross-Disciplinary Applications

Students felt the 3D printed models and the workshop had potential to be expanded across disciplines in medicine, and into new audiences. Students felt this “*could be applied to any kind of anatomy teaching whether they are pathological or normal*”, and listed examples including abdominal pathology “*intussusception and malrotation and duodenal atresia*”, fractures, “*brain lesions – like being able to see midline shift or mass effect lesions and how they’re going to compress different stuff*”, spinal malformation, and “*surgical anomalies like particularly the inguinal scrotal issues*”. In addition, students felt the audience could be expanded to include “*junior doctors even, if you were coming into an emergency rotation in areas where you might have kids presenting like this, it would be a good thing to do*”.

## Discussion

This study found that medical students were overwhelmingly positive about the inclusion of 3D printed models in the teaching of CHD. Ninety-seven percent of questionnaire respondents agreed that the self-directed TEL workshop using 3D printed models of CHD was a valuable learning experience, demonstrating widespread user satisfaction. Sub-components of the workshop that were most favourably reviewed overall included the physical 3D printed models, the clinical and case-based learning structure, and the opportunity for peer collaboration. Close to 60% of students strongly agreed that the physical models were useful and that the workshop encouraged peer collaboration, and improved their knowledge of both simple and complex CHD. These insights from the questionnaire were complemented by focus group discussions that demonstrated that the workshop guided students from a position of relative inexperience through tactile, collaborative, online-guided case-based explorations of congenital heart defects, with outcomes including perceived clinical relevance, increased understanding, and confidence in future teaching. Opportunities were identified for expansion of 3D printed model based learning.

Previously, Stunden et al. [33] published their learning outcomes following development of an optional e-learning course for first year medical students using 3D models of CHD. Medical students’ knowledge regarding CHD significantly improved on pre–/post–multiple choice questions, and the majority (88%) were highly motivated to learn with the course structure. Free-text responses suggested an enthusiastic uptake within the medical student body; however, the quantitative analysis suggesting improved knowledge acquisition was limited by the study’s use of identical pre-/post-course test questions, and a post-course test median score of 100% which represents both a response bias and a ceiling effect. Whilst we have not assessed academic performance regarding knowledge of CHD in the present study, we have demonstrated high-level acceptability of this e-learning resource and the 3D printed models. Additionally, students surveyed by Stunden et al. [33] raised concerns about social isolation in independently completing the module asynchronously online, rather than with peers, which is relevant given that students completing our workshop strongly rated the collaborative and peer-supported learning as a valuable feature. This suggests it may not be as effective to wholly digitalise pathology teaching, even with online 3D models.

Students reported increased confidence following the workshop with their knowledge about both simple and more complex CHD, and improved self-efficacy regarding their capacity to recognise the signs of CHD. Self-efficacy is a key concept in Bandura’s social cognitive learning theory, insofar as a student’s confidence in their capacity to achieve desired goals contributes to their competence in utilising their skills within their environment to do so [29]. It has previously been shown that increased self-efficacy relates to increased competence in practical anatomy and clinical examination skills [34, 35], though this has been an inconsistent finding [36, 37]. Particularly in high-stakes and complex areas of medicine, such as CHD, reducing student disinterest and disempowerment and increasing motivation and confidence in identifying signs of CHD are likely to stimulate further interest in the subject. Indeed, Bandura’s concept of self-efficacy suggests that even a slight over-estimation of one’s abilities increases an individual’s capacity to increase effort and persistence during difficult tasks [38]. Low self-efficacy is self-limiting, insofar as an individual perceives themselves as unable and likely to fail, and thus is more likely to cease efforts in the face of struggle [39]. The strongest contribution to increased self-efficacy is enactive mastery — the capacity for an individual to demonstrate to themselves that they are competent, and the self-directed case-based discovery of complex CHD with 3D models is an example of this. Enactive mastery leads to more sustained and substantial self-efficacy, compared to conventional didactic teaching, vicarious experiences (e.g. watching another student interpret the model) or verbal persuasion (e.g. facilitator delivered feedback regarding the student’s performance). This is significant as children with CHD are increasingly surviving into adulthood [11], and thus there is a need for increased generalist understanding of CHD beyond paediatric cardiac services.

Since its inception, our workshop has been expanded horizontally for teaching graduate paediatric nurses, and vertically to second year medical students. This demonstrates its scalable and reproducible design. We have re-printed the smallest models in double scale to improve appreciation of complex anatomical detail. Whilst the cost of 3D printed materials is likely to cheapen over time, and 3D printed models are more durable than waxed or plastinated cadaveric specimens, there are educational concerns that they may be perceived as less realistic than cadaveric specimens. This perception was not evident in our focus groups, and although we did not directly compare 3D models with cadaveric specimens, we have previously demonstrated equivalent medical student usage of both cadaveric and 3D printed models when given the option of both [26]. The uptake of the workshop in peripheral and rural clinical schools has increased educational equity by facilitating access to these priceless learning resources. By building a digital learning resource around the use of the 3D models, a workshop was built that could be exported to different settings and facilitated at all sites without the requirement for specialist paediatric cardiology expertise. This resource also empowers students to revise and consolidate their learning following the workshop. We have demonstrated the possibility of responding to a global call to action to preserve these specimens that are at risk of degradation over time and build a library of 3D printed congenital heart defects [40] that can be 3D printed at any location. In doing so, this study builds on previous evidence of the feasibility of 3D printed models of CHD [6, 7, 14], by demonstrating that they are an efficacious and acceptable learning aid which students are excited to learn from, that satisfies different learning preferences.

Future educators and researchers should be aware that students desire a range of sizes of 3D printed models (rather than making them universally bigger and easier), and should consider the possibility of colour-coding defects to enhance understanding, which other groups have trialled previously [3]. Students agreed least strongly with the statement that the digital models were intuitive to use, which suggests ongoing work is required to integrate the digital models to maximise their ease of use for all students. This may require improvements in relation to the software used to manipulate the online models, the labelling, and the structured exploration via TEL.

Limitations of the present study include the possibility of selection bias, where students who had a positive experience with the workshop may have been more likely to submit completed questionnaires or participate in the focus groups. This is mitigated against by evidence of occasional negative feedback amongst those surveyed and that there was no formal incentive for questionnaire completion to induce a response bias. Additionally, social desirability bias may have complicated students’ questionnaire feedback and focus group participation, although the focus group facilitators were not involved in the delivery of the workshop to the students who participated in the focus groups. As the workshop directly replaced the previous workshop due to COVID-19 restrictions, there was no opportunity to compare our workshop to the existing tutorial using cadaveric specimens. In addition, as the material was considered examinable, we were not able to provide the learning material to one group and deny it to another for the sake of comparison. Future studies may be able to directly relate the use of 3D printed models of CHD to academic performance, and compare the use of exclusively digital with hand-held 3D models or cadaveric specimens.

## Conclusions

3D printed models of CHD are efficacious learning aids in the setting of a collaborative case-based TEL module. Their use is associated with widespread medical student satisfaction and high levels of resulting self-efficacy in recognising and understanding simple and more complex CHD. The self-directed nature of the online module, which guides learning as students work through the unfolding clinical cases and explore the 3D printed models, reduces the need for specialist facilitation and encourages peer collaboration. 3D printing has the potential to facilitate scalable and equitable education innovations in the setting of CHD and other congenital anomalies.

### Electronic Supplementary Material

Below is the link to the electronic supplementary material.

Supplementary file1 (PDF 60 KB)

Supplementary file2 (DOCX 23 KB)

## Data Availability

The dataset generated during and analysed during the current study is available from the corresponding author on reasonable request.
