# Supplementary material for: An Opportunity to See the Heart Defect Physically: Medical Student Experiences of Technology-Enhanced Learning with 3D Printed Models of Congenital Heart Disease
Source: Med Sci Educ. 2023 Aug 1;33(5):1095–107. doi: 10.1007/s40670-023-01840-w (PMC10597946; doi:10.1007/s40670-023-01840-w)
Supplement: Supplementary file 1 — Supplementary file1 (PDF 60 KB) [file 40670_2023_1840_MOESM1_ESM.pdf]

THE UNIVERSITY OF SYDNEY

Paediatric Cardiac Pathology Post-Workshop Questionnaire

We invite you to comment on the paediatric cardiac pathology workshop you recently participated in. This incorporates the online module, physical 3D-printed hearts and pre-lecture materials. This is completely voluntary. Submission of your completed questionnaire constitutes your consent for your responses to be used in the research study.

Age: __________ **Gender**: ____________
Where did you participate in the workshop? (*please circle*) CHW / Dubbo / Orange / Lismore

Please rate your agreement with the following:	Strongly disagree			Neutral			Strongly agree
The workshop was a valuable learning experience	1	2	3	4	5	6	7
The case-based learning format was useful for my learning	1	2	3	4	5	6	7
Exploration of clinical aspects of the cases was useful for my learning	1	2	3	4	5	6	7
The interactive online 3D heart model was useful for my learning	1	2	3	4	5	6	7
The physical 3D-printed heart models were useful for my learning	1	2	3	4	5	6	7
Use of the physical 3D-printed heart models improved my understanding of congenital cardiac pathology	1	2	3	4	5	6	7
Navigation of the online components of the module was logical	1	2	3	4	5	6	7
Use of the interactive online 3D heart model was intuitive	1	2	3	4	5	6	7
The level of technological skill required to use the online module was appropriate	1	2	3	4	5	6	7
The workshop encouraged interaction and collaboration with my peers	1	2	3	4	5	6	7
The workshop enabled me to improve my knowledge about simple congenital heart disease (e.g. ASDs, VSDs)	1	2	3	4	5	6	7
The workshop enabled me to improve my knowledge of complex congenital heart disease (e.g. TOF)	1	2	3	4	5	6	7
I feel more confident in my knowledge of congenital heart disease than before the workshop	1	2	3	4	5	6	7
I feel more confident in my ability to recognise signs of congenital heart disease than before the workshop	1	2	3	4	5	6	7

Please turn over

How could the quality of the workshop (including the online module, 3D-printed hearts and pre-lecture materials) be improved?

What would you keep the same?

What would you change?

Overall, what elements of this workshop most contributed to your excitement and engagement as a learner (e.g., online module, 3D-printed hearts, pre-lecture materials)?

What could have been done to improve your engagement?

Thank you for your time
