# Supplementary material for: An Opportunity to See the Heart Defect Physically: Medical Student Experiences of Technology-Enhanced Learning with 3D Printed Models of Congenital Heart Disease
Source: Med Sci Educ. 2023 Aug 1;33(5):1095–107. doi: 10.1007/s40670-023-01840-w (PMC10597946; doi:10.1007/s40670-023-01840-w)
Supplement: Supplementary file 2 — Supplementary file2 (DOCX 23 KB) [file 40670_2023_1840_MOESM2_ESM.docx]

**Focus Group Questions**

*Participants will be thanked for joining the focus group and reminded that their participation is completely voluntary and they may withdraw at any time without consequence. Confirmation of consent forms will be undertaken. Participants will be informed prior to the commencement of audio recording.*

Some medical students (and graduate doctors) find paediatric cardiology challenging – how have you found teaching on paediatric cardiology during the CAH block? *What was your previous exposure to the subject area?*

What do you think is the value of learning about congenital heart disease? *Do you anticipate needing to know about congenital heart disease in your future careers? Where might you need this knowledge?*

Reflecting on your paediatric cardiology teaching this block – what were the most difficult concepts to understand?

In week 5, you completed a 30-minute case-based workshop on congenital heart disease incorporating an online module and 3D printed models of heart disease – how did you find this? *What did you enjoy? What did you find difficult or confusing?*

How did you find the workshop compared to other modes of learning you have encountered in your studies? Was it engaging? *Too practical? Too theoretical?*

Specifically regarding the 3D-printed hearts – how did you find using and holding the hearts? *Do you feel these models are a good representation of human anatomy and pathology? Did you feel comfortable holding or manipulating the models? Did they seem fragile or breakable? Were you confused about how best to learn from the 3D-printed models?*

Specifically regarding the online module - were there any specific features you found helpful? *Were there any specific features you found confusing or unhelpful?*

How did you find the experience of being guided to explore the physical models by the digital prompts? *Did you find the ‘auto-orientation’ feature useful to help orient the model and correctly identify anatomy? How did you find using the zooming feature? How did you find the labels? Did you turn them off? If so, how did that affect your learning?*

Which 3D model did you prefer to explore – the online interactive model or the physical 3D-printed heart? *Why did you prefer this model? Did you feel that the two models were complementary, or redundant? Did you use each in a similar way, or where there differences?*

Did you review the online pre-learning (fetal and transitional circulation video, embryology video) prior to the workshop? *Did this help your preparation? Did you feel this was adequate preparation? What other resources would you have found helpful?*

Have the online learning module & 3D-printed hearts helped you to understand congenital heart disease? *Do you feel more confident in your knowledge about congenital heart disease? Do you feel more confident in your ability to recognise signs of congenital heart disease? How do you feel the online learning module & 3D-printed hearts have helped you prepare for being a junior doctor?*

Are there other areas of your medical education for which you would find it helpful to have a 3D-printed anatomical model of disease processes?

Do you have any suggestions for improving this workshop?
